# Detection of antimicrobial resistance via state-of-the-art technologies versus conventional methods

**DOI:** 10.3389/fmicb.2025.1549044

**Published:** 2025-02-25

**Authors:** Ayman Elbehiry, Eman Marzouk, Adil Abalkhail, Moustafa H. Abdelsalam, Mohamed E. A. Mostafa, Mazen Alasiri, Mai Ibrahem, Abousree T. Ellethy, Abdulaziz Almuzaini, Sahar N. Aljarallah, Akram Abu-Okail, Naif Marzook, Satam Alhadyan, Husam M. Edrees

**Affiliations:** ^1^Department of Public Health, College of Applied Medical Sciences, Qassim University, Buraydah, Saudi Arabia; ^2^Department of Physiology, Faculty of Medicine, University of Tabuk, Tabuk, Saudi Arabia; ^3^Department of Anatomy, Faculty of Medicine, University of Tabuk, Tabuk, Saudi Arabia; ^4^Department of Pharmacy, Armed Forces Hospital, King Abdul Aziz Naval base in Jubail, Jubail, Saudi Arabia; ^5^Department of Public Health, College of Applied Medical Science, King Khalid University, Abha, Saudi Arabia; ^6^Division of Biochemistry, Department of Basic Oral Sciences and Dental Education, College of Dentistry, Qassim University, Buraydah, Saudi Arabia; ^7^Department of Veterinary Preventive Medicine, College of Veterinary Medicine, Qassim University, Buraydah, Saudi Arabia; ^8^Department of Pharmacy sciences, College of Pharmacy, AlMaarefa University, Riyadh, Saudi Arabia; ^9^Department of Pathology and Laboratory Diagnosis, College of Veterinary Medicine, Qassim University, Buraydah, Saudi Arabia; ^10^Department of Emergency Medicine, King Fahad Armed Forces Hospital, Jeddah, Saudi Arabia; ^11^Department of Environmental Health Administration, Health Services, Ministry of Defense, Riyadh, Saudi Arabia

**Keywords:** antibiotic resistance, detection, contemporary technologies, genomics, microbial community

## Abstract

Antimicrobial resistance (AMR) is recognized as one of the foremost global health challenges, complicating the treatment of infectious diseases and contributing to increased morbidity and mortality rates. Traditionally, microbiological culture and susceptibility testing methods, such as disk diffusion and minimum inhibitory concentration (MIC) assays, have been employed to identify AMR bacteria. However, these conventional techniques are often labor intensive and time consuming and lack the requisite sensitivity for the early detection of resistance. Recent advancements in molecular and genomic technologies—such as next-generation sequencing (NGS), matrix-assisted laser desorption ionization-time of flight mass spectrometry (MALDI-TOF MS), lateral flow immunoassays (LFIAs), PCR-based diagnostic methods, and CRISPR-based diagnostics—have revolutionized the diagnosis of AMR. These innovative approaches provide increased sensitivity, reduced turnaround times, and the ability to identify genetic resistance mechanisms. This review seeks to examine the advantages and disadvantages of both emerging technologies and traditional methods for detecting AMR, emphasizing the potential benefits and limitations inherent to each. By understanding the strengths and limitations of these technologies, stakeholders, including researchers, healthcare professionals, regulatory agencies, health authorities, financial managers, and patients, can make informed decisions aimed at preventing the emergence and dissemination of antibiotic-resistant strains, thereby ultimately increasing patient safety.

## 1 Introduction

Since penicillin was discovered, antimicrobial drugs have greatly advanced medicine and community health (Breijyeh and Karaman, 2023; Gould, 2016; Shanmugakani et al., 2020; Zaffiri et al., 2012). They have saved millions of lives and controlled communicable diseases, which were leading causes of mortality before their introduction (Aminov, 2010; Aminov, 2017). The development of antimicrobials has been a key advancement in healthcare (Brown and Wright, 2016; Yamin et al., 2023). However, new antibiotic-resistant bacteria frequently emerge shortly after their introduction (Hu et al., 2020; Lobanovska and Pilla, 2017). Penicillin, recognized as the first beta-lactam antibiotic, fundamentally transformed the management of infectious diseases and facilitated the development of subsequent antibiotic classes, including sulfonamides and aminoglycosides, such as streptomycin and streptothricin. Since its initial discovery, antimicrobial research has progressed significantly, leading to the emergence of novel classes of antimicrobial agents (Ventola, 2015). Currently, a diverse array of antibiotics are commonly used, including modified beta-lactams, cephalosporins, tetracyclines, fluoroquinolones, and aminoglycosides. Nevertheless, the increasing incidence of antibiotic resistance has compelled researchers to investigate natural compounds as potential alternatives to address the therapeutic challenges that have arisen since the mid-20th century (Hunt and Kates, 2024). Although traditional antimicrobials are common in agriculture, new antimicrobial drugs are scarce (Miller et al., 2022). Misadministration of antimicrobials in humans and animals (Caneschi et al., 2023) contributes to an increase in the prevalence of antibiotic-resistant bacteria (Golding et al., 2019; Shanmugakani et al., 2020; Singer et al., 2016; Tangcharoensathien et al., 2018). Factors such as healthcare and environmental influences facilitate this spread (Ahmad et al., 2021; Bengtsson-Palme et al., 2018; Serweciñska, 2020), posing a significant threat to public health and the economy in both developed and developing countries (Naylor et al., 2018; Weldon and Hoffman, 2023).

Antimicrobial resistance is a major global health threat in the 21st century, requiring urgent action as per World Health Organization (WHO) recommendations (Vasala et al., 2020). This resistance has made common diseases, such as respiratory and cardiovascular illnesses, more prevalent and difficult to treat (Bassetti et al., 2017). In 2019, it was responsible for approximately 1.27 million deaths and contributed to an additional 4.95 million fatalities worldwide (Murray et al., 2022). Antibiotic-resistant microbes cause more than 33,000 deaths annually in Europe (Antoñanzas and Goossens, 2019; Peñalva-Moreno et al., 2022) and affect more than three million people in the Unites States., resulting in more than 35,000 deaths each year (Church and McKillip, 2021). In India, more than 50,000 infants die from sepsis due to resistant bacteria, with an infant dying every 9 min from such infections (Subramaniam and Girish, 2020).

In 2019, the number of deaths from methicillin-resistant *Staphylococcus aureus* (MRSA) surpassed 100,000 (Alghamdi et al., 2023). The WHO’s Global Antimicrobial Surveillance reported over 500,000 confirmed infections from bacteria with extensive AMR. ESKAPE pathogens—*Enterococcus faecium*, *Staphylococcus aureus* (*S. aureus*), *Klebsiella pneumoniae* (*K. pneumoniae*), *Acinetobacter baumannii* (*A. baumannii*), *Pseudomonas aeruginosa* (*P. aeruginosa*), and *Enterobacter* spp.—are the most commonly isolated resistant organisms in hospital environments (Miller and Arias, 2024). Healthcare-associated infections (HAIs) constitute a major health issue, affecting 5% to 10% of hospitalized patients and costing the United States healthcare system approximately USD 4 billion annually. A 2011 survey revealed that 4% of hospitalized patients had an HAI (Magill et al., 2018). AMR contributes to the increase in HAIs, complicating cost management (Salam et al., 2023b). Patients with drug-resistant pathogens face longer hospital stays and higher costs (Fongang et al., 2023). Strict infection prevention protocols, judicious antibiotic use, and monitoring for resistant bacteria are crucial for reducing costs and improving patient safety.

Antibiotic stewardship programs (ASPs) are essential for reducing global antibiotic resistance (Majumder et al., 2020). They promote appropriate antimicrobial use by identifying the most effective agents, durations, doses, and administration methods, thus minimizing side effects and costs (Ababneh et al., 2021). Successful ASPs require leadership, hospital management support, skilled infectious disease clinicians, ongoing education, and interdisciplinary collaboration (Baraka et al., 2019). The WHO is working with various organizations to develop strategies that raise awareness of AMR (World Health Organization, 2014). These strategies aim to reduce transmissible diseases through precautionary measures, improved antibiotic therapy, novel treatments, and enhanced antimicrobial drug efficacy (World Health Organization, 2014). Despite significant advancements in rapidly detecting antibiotic-resistant bacteria (Yamin et al., 2023), the effectiveness of antibiotics is declining (Chinemerem Nwobodo et al., 2022). Microbial infections are often hard to identify, causing treatment delays (Cansizoglu et al., 2019; Giordano et al., 2018). As a result, doctors may need to start antimicrobial treatment before a full evaluation, which can worsen the patient’s condition and contribute to AMR (van Belkum et al., 2019). There is an urgent need for rapid, accurate, and affordable tests to detect AMR (Salam et al., 2023b), enabling better-targeted medications and reducing the time for antimicrobial susceptibility testing (AST) (Cansizoglu et al., 2019). Screening is crucial in managing AMR (Khan et al., 2019).

Microbiological investigations often use phenotypic methods to assess infection sensitivity to antibiotics (Maugeri et al., 2019; van Belkum et al., 2020). Although these techniques are cost-effective and standardized, they can be labor intensive, causing treatment delays (Shin et al., 2019; Syal et al., 2017) that negatively impact patient management (Khan et al., 2019). Delayed antibiotic treatment is associated with higher mortality rates (Pak et al., 2023) and longer hospital stays (Nauclér et al., 2021). Comprehensive surveillance systems are vital for reducing AMR-related mortality and morbidity, guiding treatment decisions, and developing new antibacterial medications (Cantón et al., 2023). The Study for Monitoring AMR Trends (SMART) includes nearly 500,000 isolates from over 200 sites in more than 60 countries, addressing evolving medical needs (Cantón et al., 2023). SMART data have identified emerging resistance risks and informed clinical guidelines, making the database accessible to clinicians and researchers worldwide, particularly in resource-limited countries. The development of rapid tests for antibiotic susceptibility aims to improve patient treatment and manage AMR (Datar et al., 2022; Gulumbe et al., 2022; Zakhour et al., 2023) by quickly identifying pathogenic microorganisms and their susceptibility to antibiotics.

**FIGURE 1:**
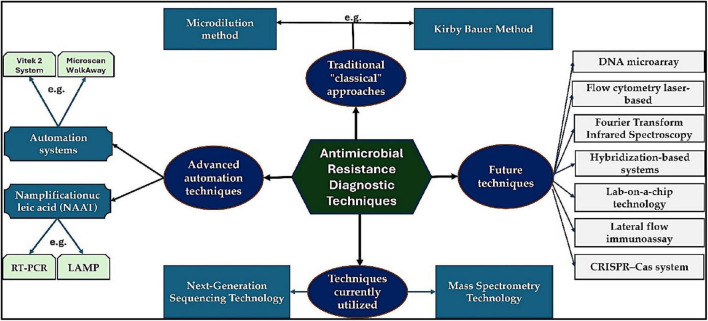
A brief overview of the various techniques and methodologies used in the current review.

This review examines both traditional and contemporary methodologies for the detection of bacteria that have acquired resistance to antimicrobials. The objective of this review was to improve our understanding of the effectiveness of AST in assessing the susceptibility of bacteria to various antibiotics. We provide a thorough analysis of the advantages and limitations associated with current AST methods while also highlighting significant advancements in point-of-care testing. Additionally, we present forecasts regarding the most promising technologies expected to emerge in the future for ASTs. Figure 1 provides a summary of the conventional and modern technologies discussed in this review. To ensure the relevance of the included research, only articles published in English between 2002 and 2024 were considered.

## 2 Antimicrobial susceptibility: technologies of the past, present, and future

### 2.1 Traditional “classic” approaches

Organizations such as the Clinical and Laboratory Standards Institute (CLSI) and the European Committee on AST recommend phenotypic screening to identify antibiotic-resistant bacteria (Gajic et al., 2022; Maugeri et al., 2019). Traditional AST methods, including agar dilution, broth microdilution, and disk diffusion, involve exposing isolated bacteria to antimicrobial agents and observing their growth (Puttaswamy et al., 2018; Salam et al., 2023a). A key method for assessing resistance is determining the minimum inhibitory concentration (MIC) (Kowalska-Krochmal and Dudek-Wicher, 2021), which determines the lowest antibiotic concentration that inhibits bacterial growth via agar or broth. This technique mixes a standardized number of bacteria (typically 0.5 according to the McFarland standard) with antibiotic-diluted broth or agar (Wiegand et al., 2008). In brief, the MIC assay was conducted by standardizing a bacterial culture to 5 × 105 colony-forming units (CFU)/ml and exposing it to various antibiotic concentrations for 16–24 h at 37°C (Lewis and James, 2022). After the incubation period, bacterial growth was evaluated for each antibiotic concentration, and the MIC value was identified as the lowest concentration of the antimicrobial agent needed to inhibit visible growth of the bacterial strain being studied.

After incubation, bacteria may or may not grow, and the procedure is simple and inexpensive, requiring no specialized instruments. The first step in therapeutic treatment often involves measuring the MICs of antimicrobial agents against bacteria (Gajic et al., 2022). However, this technique has limitations in assessing resistance in non-cultivable yet viable microbial species, and its effectiveness depends on factors such as incubation duration, antimicrobial concentration, and initial microbial inoculum (Wiegand et al., 2008). The disk diffusion method involves placing an antimicrobial-infused disk on solid agar, creating a circular zone of inhibition that indicates bacterial growth suppression. This qualitative analysis classifies bacteria as susceptible, intermediate, or resistant. While effective for rapidly multiplying bacteria, the method has drawbacks, including low agar diffusion of some antimicrobial agents and difficulties in evaluating anaerobic and fastidious bacteria (Sejas et al., 2003). Clinical laboratories use a variety of antimicrobial susceptibility testing (AST) methods, depending on the specific equipment and range of laboratory tests they provide (Gajic et al., 2022).

The E-Test, the original gradient strip, a proprietary tool from bioMérieux, is widely used in clinical laboratories to guide antibiotic selection by indicating effective antimicrobial concentrations (Salam et al., 2023a). It combines elements of previous methods to generate MIC data via disk diffusion (Erfani et al., 2011). The E-test^®^ features strips with an exponential gradient of antibiotic concentrations and a numeric scale. While it shares time-related limitations with earlier tests, it simplifies microbial susceptibility quantification (Yamin et al., 2023) and is particularly effective for hard-to-cultivate microorganisms such as *Mycobacterium* (obligate aerobe) and *Haemophilus influenzae* (facultatively anaerobic) (Hedberg, 2005). The rapid direct E test is an effective tool for obtaining preliminary AST data within a period of 5–6 h. This method is especially beneficial when it is employed alongside phenotypic or genotypic analyses to clarify essential mechanisms of resistance (Bianco et al., 2019b). The gradient strips are used for precise quantification of resistant strains in laboratory and clinical settings because of their stable concentration gradient (Khan et al., 2019). Di Bonaventura et al. (1998) reported that E-test MICs correlated well with agar dilution and disk diffusion methods for 248 *P. aeruginosa* isolates from bladder-catheterized patients, confirming their reliability. Using the EUCAST Rapid Antimicrobial Susceptibility Testing (RAST) technique, Bianco et al. (2022b) examined 676 positive blood cultures derived from gram-negative rods and gram-positive cocci. RAST was performed within 2 h of leaving the incubator, in accordance with EUCAST recommendations. Inhibition zones were evaluated at 4, 6, 8, and 16–20 h, and the results were interpreted according to EUCAST breakpoints (version 5.1). The results after 16–20 h suggest the potential for more effective antibacterial de-escalation therapy. Despite these limitations, traditional methods for detecting AMR are crucial for identifying treatment options for antibiotic resistance. Despite their drawbacks, traditional tests are inexpensive, easy to perform (requiring no special expertise), and still widely used in hospitals around the world (Kaprou et al., 2021; Yalew, 2020).

### 2.2 Advanced automation techniques

#### 2.2.1 Automation systems

The sensitivity of optical equipment for ASTs can be enhanced with optoelectronic systems, fiber optics, microfluidics, and pH- or redox-sensitive indicator dyes (She and Bender, 2019; Trovato et al., 2022). FDA-approved programmable AST devices, such as the Sensititre™ ARIS™ 2X, Vitek 2 Compact System, Phoenix™ panel systems, and MicroScan WalkAway plus systems, significantly impact laboratory settings (Bhatnagar et al., 2023; Elbehiry et al., 2023; Fader et al., 2013; Leonard et al., 2018). These devices feature antibacterial panels for gram-positive and gram-negative infections and use redox indicators to identify bacteria and measure turbidity from multiwell plates. AliFax S.r.l. in Italy utilizes the ALFRED 60/AST system, which employs laser-light scattering technology to detect bacterial growth in liquid culture, providing susceptibility data within four to 6 h from positive blood cultures (López-Hernández et al., 2022). Broth dilution is used with AST cards containing varying antimicrobial concentrations and positive controls, allowing for analysis of MIC patterns in large microorganism clusters (Puttaswamy et al., 2018; Vasala et al., 2020).

The Microscan WalkAway system measures experimental progress via a photometer or fluorometer and can continuously monitor incubated samples (Shanmugakani et al., 2020). MicroScan panels use conventional 96-well microdilution plates for bacterial identification, typically within four hours, although slow-growing bacteria may take 6–42 h (Reller et al., 2009). Susceptibility results are available in 20 h, varying from 16.8 to 27.8 h on the basis of the bacteria type (Winstanley and Courvalin, 2011). In brief, a bacterial suspension corresponding to the 0.5 McFarland standard was prepared in saline via the direct colony suspension method. Subsequently, 5 μl of the diluted suspension was dispensed into each well. Before inoculation, the panels were checked for expiration dates, batch numbers, and packaging integrity. The inoculation was carried out via Prompt Inoculation System-D in conjunction with the RENOK system and Inoculators-D. The panels were then incubated at 35 ± 1°C for 16 h, after which readings were taken. The MIC was determined on the basis of standardized readings provided by the manufacturer. Successful antimicrobial susceptibility values were assessed and evaluated according to the European Committee on Antimicrobial Susceptibility Testing.

A pilot study in a tertiary care teaching hospital located in Rishikesh, Uttarakhand (Singh et al., 2019), in May and June 2019 used the MicroScan WalkAway 96 Plus ID/AST system and Mikrolatest MIC kit to assess colistin susceptibility in carbapenem-resistant gram-negative bacteria. The susceptibility rates were 71.4% for *A. baumannii*, 85.7% for *P. aeruginosa*, and 100% for *Acinetobacter junii*, *Acinetobacter johnsonii*, *Escherichia coli* (*E. coli*), and *K. pneumoniae*. Hernández-Durán et al. (2017) assessed bacterial susceptibility to antibiotics in hospitalized patients via the VITEK 2^®^ Compact and MicroScan WalkAway^®^ SI systems. The study included 20 gram-positive cocci, 34 g-negative rods, and 13 reference strains. Both techniques showed 90.2% concordance for gram-negative bacteria and 96.3% for gram-positive bacteria, identifying 89.5% of strains by species. VITEK 2 had a median result time of 6.5 h, whereas MicroScan took 12.5 h, indicating a significant delay.

The BD Phoenix Automated Microbiology System is a reliable tool for identifying clinical isolates in healthcare laboratories (Hirakata et al., 2005). It accurately tests most clinically relevant bacteria (Donay et al., 2004; Funke and Funke-Kissling, 2004) and detects ESBLs (Sanguinetti et al., 2003). The system analyzes 99 test panels, each with 84 wells of antibacterial agent dilutions (Yamin et al., 2023). Growth is monitored with a turbidimeter and calorimeter, enabling MIC estimation for various pathogens, including gram-negative and gram-positive bacteria, within 6–16 h (Shanmugakani et al., 2020). The Vitek system, developed in the late 1970s, automates AST and identification. After standardizing the main inoculum, all the necessary steps are performed, and the results are analyzed every 15 min for kinetic analysis (Funke et al., 1998; Ligozzi et al., 2002). The system records colorimetric, turbidity, and fluorescence signals via multichannel fluorimeters and photometers. It employs 64-well reagent cards containing diagnostic media and antimicrobial agents (Abalkhail and Elbehiry, 2022; Elbehiry et al., 2022b), allowing for 30–240 simultaneous assays for gram-negative and gram-positive bacteria within 4–10 h (Shanmugakani et al., 2020). The protocol for the Vitek system for the detection of AST is as follows: 2–3 fresh colonies of the tested bacteria were transferred to a 5 mL tube of sterile physiological sodium chloride solution. The 0.5 McFarland bacterial mixture was then diluted to 1.5 × 10^7 CFU/mL in 0.45% saline (0.50–0.63) via the DensiChek device (BioMérieux, Marcy l’Etoile, France). After filling, sealing, and inserting the AST cards into the VITEK 2 device (VITEK^®^ 2, BioMerieux, France), they were incubated and read. The results were analyzed in accordance with the guidelines established by the CLSI.

At the Prince of Wales Hospital in Hong Kong, China, Ling et al. (2003) conducted a study on bacterial identification and susceptibility to various antibiotics from July 21 to December 15, 2002. Using the VITEK 2 system, they performed direct identification and susceptibility testing on an aerobic bottle from a positive blood culture set. The results of the susceptibility testing were available 3.3 h postincubation, while identification reports were also provided 3.3 h after incubation. A unique method for assessing antibiotic resistance involves detecting volatile organic molecules in the headspace of bacterial cultures. Therefore, Bianco et al. (2024) evaluated the VITEK^®^ REVEAL system on 128 positive blood cultures, including 95 *Enterobacterales*, 21 *P. aeruginosa*, and 12 *A. baumannii* complex samples. The study compared 22 antimicrobials with reference techniques across 2,220 strain-antibiotic combinations, revealing 1,091 resistant pairings (48.7%). The categorical agreement and essential agreement rates were 97.6 and 97.7%, respectively. In the ESBL phenotype screening test, positive, indeterminate, and negative results were found for 13.7, 32.6, and 27.4% of the *Enterobacterales* isolates, respectively, with 100% concordance with the reference technique. This system effectively assesses antimicrobial susceptibility in major gram-negative species from positive blood cultures, delivering results in under 8 h. Charnot-Katsikas et al. (2018) evaluated the Accelerate Pheno system at the University of Chicago Medicine for identifying bacterial and yeast species and conducting AST. When the blood culture results were compared with those of routine care within 0–8 h of growth detection, the system showed 95.6% sensitivity and 99.5% specificity for identification, with 95.1% agreement in essential susceptibility tests and 95.5% agreement in categorical susceptibility tests. The accelerated Pheno system reduces the susceptibility testing time by an average of 41.86 h, enabling quicker delivery of clinically relevant information.

Traditional phenotyping techniques are slow because of the multiple stages of plating and cultivation, making them unsuitable for rapid results (Elbehiry et al., 2017; Elbehiry et al., 2022a). Researchers have developed genetic methods to detect AMR to address this issue (Anjum et al., 2018; Sahoo et al., 2023). The term “molecular diagnostics” was introduced by Pauling et al. (1949). Genetic methods, such as multiplex targeting, offer advantages over phenotypic testing in identifying antibiotic resistance genes (Yamin et al., 2023). These methods are useful when sensitivity breakpoints are unknown and can use non-purified samples, allowing for faster responses to new resistance components (Vasala et al., 2020). Genetic approaches have limitations, including low sensitivity and narrow scope, and often fail to overlook many AMR genes (Gajic et al., 2022). Additionally, molecular diagnostics can be costly (Singh et al., 2021). However, advancements in techniques such as nucleic acid amplification and hybridization are improving detection (Bai et al., 2023). Overall, molecular methods are effective and sensitive for identifying AMR genes (Kaprou et al., 2021; Rentschler et al., 2021).

#### 2.2.2 Nucleic acid amplification tests (NAATs)

Genetic evaluations, such as virulence genotyping and multilocus sequence typing, are more suitable for outbreak investigations than are ASTs (Vasala et al., 2020). These methods require large amounts of pure nucleic acids, making them impractical for rapid diagnosis. In contrast, molecular beacon systems and hybridization-based strategies are effectively integrated into NAATs (Yamin et al., 2023). The CDC states that commercial NAATs are used mainly in hospitals, health agencies, and private labs (Ling et al., 2008). Many low-income countries still rely on in-house PCR tests, which cost $15 each (Roos et al., 1998). These nations often have the highest case numbers, limiting their access to expensive technologies. In the last two decades, NAATs have become essential for detecting microorganisms, greatly assisting in diagnostic testing and research on infectious diseases (Apfalter et al., 2005). Global migration, climate change, urbanization, and the ongoing pandemic emphasize the significance of NAATs. Although NAATs are known for their high accuracy and sensitivity, their centralized structure and dependence on qualified personnel can impede effective pathogen management during emergencies (Narasimhan et al., 2023).

Nucleic acid amplification tests are highly effective for identifying microbial species, especially when combined with a strong molecular test (Patel, 2022). Recent diagnostic panels developed by companies such as BioMérieux, Qiagen, and Becton Dickinson can identify genes related to antibiotic resistance (Trotter et al., 2019; Vasala et al., 2020) and provide clinically relevant data even when antimicrobial treatment is not needed (Vasala et al., 2020). Among drug-resistant pathogens, only a few, such as *Neisseria gonorrhea*, *Chlamydia trachomatis*, and *Mycoplasma*, have shown a certain degree of resistance (Unemo and Jensen, 2017). Identifying genes linked to antibiotic-resistant bacteria is promising but does not confirm resistance (Salyers and Amabile-Cuevas, 1997). A key limitation of NAATs is their inability to specify MICs or recommend antibiotics (Yu et al., 2023). Nonetheless, NAATs can be easily adapted to address resistance factors and emerging infections (Bang et al., 2023). Techniques such as isothermal amplification, polymerase chain reaction (PCR), multiplex PCR, reverse transcriptase PCR (RT-PCR), and quantitative PCR (qPCR) can be used to identify antibiotic resistance genes.

The isothermal DNA amplification technique is a breakthrough in molecular biology that eliminates the need for thermal cycling in conventional PCR (Lee et al., 2019; Zhu et al., 2020). Techniques such as transcription-mediated amplification (TMA) and loop-mediated isothermal amplification (LAMP) have been developed (Glökler et al., 2021; Srivastava and Prasad, 2023), leading to next-generation genetic diagnostics that better serve patients (Lee et al., 2019). Isothermal methods bypass thermocycling, reducing analysis times and energy consumption (Oliveira et al., 2021), and alternative heat control methods have rendered thermocyclers obsolete (Zou et al., 2020). Additionally, isothermal amplification offers higher rates of amplification and specificity than PCR does (Zanoli and Spoto, 2012). A recent study investigated the efficacy of the Amplex eazyplex^®^ LAMP assay for rapidly identifying *A. baumannii* and its predominant acquired carbapenemases directly from blood culture bottles in less than 30 min (Comini et al., 2021). The findings of this study indicate that the Amplex eazyplex^®^ LAMP assay is proficient at detecting *A. baumannii* and carbapenemases associated with carbapenem resistance. In numerous international airports, hospitals, and testing facilities globally, LAMP has emerged as a viable alternative to qPCR (Subsoontorn et al., 2020). However, these methods face challenges, including less effective multiplexing due to experimental complexity (Dhama et al., 2014) and the need for more primers in complex reactions.

Polymerase chain reaction is an *in vitro* technique used to amplify DNA and RNA sequences exponentially (Bej et al., 1991). False positives may arise from infections or cross-reactivity with similar microorganisms (Hahn et al., 2020). Therefore, quality control measures for PCR assays, particularly in diagnostics, start with established laboratory practices that reduce contamination and increase reproducibility (Kitchin and Bootman, 1993). A significant advantage of PCR over traditional culture methods is its ability to amplify genes from uncultivable or dying organisms (Adzitey et al., 2013). Overall, PCR is effective for tracking AMR infections, identifying known resistance genes, such as *mecA* (methicillin resistance) in *S. aureus* (Abalkhail and Elbehiry, 2022), *blaSHV* (beta-lactam resistance) in Enterobacteriaceae (Zorbozan and Kimiran, 2022), *tetM* (tetracycline resistance) (Domínguez-Pérez et al., 2018), and *vanA* (vancomycin resistance) in *Enterococcus* (Wagner et al., 2023; Wardal et al., 2023). An earlier investigation assessed the effectiveness of ELITe MGB assays in detecting the main carbapenemase and ESBL genes and mec genes associated with *S. aureus* in blood cultures within a timeframe of less than three hours (Bianco et al., 2019a). The findings of the study indicated a concordance between genotypic and conventional phenotypic outcomes. Boattini et al. (2021) conducted a study on molecular tests to enhance antibiotic treatment for critically ill patients with carbapenemase- and/or CTX-M-producing pneumonia. They examined 197 bronchoalveolar lavage (BAL) samples for carbapenem-resistant Enterobacteriaceae (CRE) and ESBL using ELITe MGB^®^ assays and compared them to standard culture methods. Twenty (10.2%) strains tested positive for *bla*_*KPC–like*_ genes, and twelve (6.1%) strains tested positive for *bla*_*CTX–M–like*_ genes. The CRE ELITe MGB Kit demonstrated a positive predictive value (PPV) of 85% (95% CI: 64.9–94.6) and a negative predictive value (NPV) of 100%. The ESBL ELITe MGB Kit had a PPV of 75% (95% CI: 49.4–90.2) and an NPV of 100%.

Multiplex PCR may outperform conventional and quantitative PCR in increasing the possibility of cross-contamination during multiplexing (Adler et al., 2008). The use of different primers in a solution blend allows the identification of various bacteria in a single test run, reducing costs and time (Pishnian et al., 2019). Multiplex PCR can effectively identify specific mutations in AMR genes (Banerjee and Patel, 2023; Chahorm and Prakitchaiwattana, 2018) if one primer interacts with the mutation points. It has been developed to quickly and cost-effectively identify AMR genes in infections and multiple organisms in patient samples (Sigmund et al., 2019). However, information on its effectiveness for detecting antibiotic resistance indicators in healthcare settings is limited (Sigmund et al., 2020).

Reverse transcriptase polymerase chain reaction converts RNA into complementary DNA (cDNA) and amplifies it via PCR. This method is efficient, responsive, and reliable (Adzitey et al., 2013; Rodrigues et al., 2018). cDNA produced from RNA is free of impurities such as proteins that can skew analyses, allowing for precise identification with primers. RT-PCR also detects viable microbes, including antibiotic-resistant organisms (Rajapaksha et al., 2019), and is highly specific for replicating cells. A study was conducted at the Hospital of Tropical Diseases in Ho Chi Minh City by Dung et al. (2024). They collected tracheal aspirates and sputum from patients with lower respiratory tract infections to identify and detect AMR genes for various bacteria. The Microbiology Department at the hospital used RT-PCR to identify six bacterial pathogens and their AMR genes. The results revealed that this technique is rapid, straightforward, and highly specific. Treating infections caused by antibiotic-resistant microorganisms, especially those with high AMR gene concentrations, is challenging. Real-time PCR and reverse transcription-PCR are currently used together to evaluate gene expression variations.

### 2.3 Techniques currently being utilized

#### 2.3.1 Next-generation sequencing (NGS) technology

The NGS method has been developed for sequencing DNA and RNA and detecting mutations and variants (Qin, 2019; Satam et al., 2023). NGS can sequence thousands of genes or entire genomes in a short time (Goodwin et al., 2016). Integrating DNA and RNA next-generation sequencing (NGS) is the most effective strategy for understanding and adapting to the evolving resistance mechanisms to targeted therapies (Reita et al., 2021). NGS is particularly recognized for evaluating organisms with slow progression and unusual resistance patterns, such as highly resistant *Mycobacterium tuberculosis* isolates (Iketleng et al., 2018). Since the 1995 sequencing of *Haemophilus influenzae* type B (Fleischmann et al., 1995), advances in genomic technology have transformed infection prevention, control, and healthcare delivery (Purushothaman et al., 2022). Key methodologies include whole-genome sequencing (WGS), which analyzes the complete genome of a single bacterial colony, and metagenomics, which examines microbial communities in a sample without considering the cultural background (Chiu and Miller, 2019; Eyre, 2022). The cost of nucleotide sequencing has decreased significantly since the early 21*^st^* century (Brown et al., 2021; Dymond et al., 2020) owing to advancements in sequencing capabilities, affordable technologies, improved laboratory automation, and standardized procedures (Perez-Sepulveda et al., 2021). WGS is now used as a diagnostic tool in various laboratories for medical microbiology diagnosis and monitoring (Bonsall et al., 2020; Smetana and Brož, 2022; Van Belkum and Rochas, 2018). Its ability to quickly identify resistance factors has influenced treatment strategies (Iketleng et al., 2018).

However, recent challenges have limited the widespread use of NGS in resistance screening (Ávila-Ríos et al., 2020). NGS faces challenges such as high costs and limited user-friendly bioinformatics systems (Sherry et al., 2013). Nevertheless, high-quality sequencing can be achieved within days to a week (Sherry et al., 2013). NGS is anticipated to replace standard cultivation methods in the medium or long term (Femerling et al., 2023) and serve as a practical alternative for routine microbiological testing to identify bacterial infections and predict antibiotic susceptibility (Dunne et al., 2012). Additionally, NGS has the potential to provide both typing results and the ability to detect resistance genes in a single experiment (Veenemans et al., 2014). Many innovative NGS applications have recently transitioned from research to clinical use (Di Resta and Ferrari, 2018), including cell-free DNA analysis in prenatal testing, circulating tumor DNA testing, HLA typing, microbial analysis (Yohe and Thyagarajan, 2017), RNA sequencing, and methylation analysis. Despite ongoing challenges, these technologies are increasingly employed for therapeutic purposes.

Whole-genome sequencing antibiotic susceptibility testing is a rapid and accurate method for detecting antibiotic resistance (MacLean et al., 2020). The correlation between genotypes and clinical phenotypes is not always accurate, for example, mechanisms involving inducible resistance, gene expression and regulation, posttranslational modifications or combinations thereof. Consequently, rapid phenotypic testing and growth-based susceptibility testing are expected to be required initially to confirm an NGS result (Ellington et al., 2017). Most resistance strategies involve multiple genes and complex cellular networks, which are not well understood. WGS provides a novel approach to studying these systems by examining the entire genomes of microorganisms in clinical settings. A systematic review by Yusof et al. (2022) revealed that WGS is commonly used to detect variants in colistin resistance genes among *K. pneumoniae* isolates. WGS significantly advances AMR research by analyzing all contributing factors and identifying relationships between resistance genes and their host elements. WGS enables genome-wide studies of multiple genes and alterations (De Been et al., 2015; Kohl et al., 2014; Lin et al., 2022). Katiyar et al. (2020) used WGS to explore resistance genes and their symptom correlations, aiding in targeted treatment selection. These findings revealed that fluoroquinolone-resistant isolates presented changes in the *gyrA*, *gyrB*, *parC*, and *parE* genes. By predicting resistance polymorphisms and their phenotypic relationships, WGS enhances understanding and facilitates efficient identification of AMR correlates, allowing for antibiotic use on the basis of genetic factors (Wan Makhtar et al., 2021).

#### 2.3.2 Mass spectrometry (peptide fingerprinting analytical technique)

**FIGURE 2:**
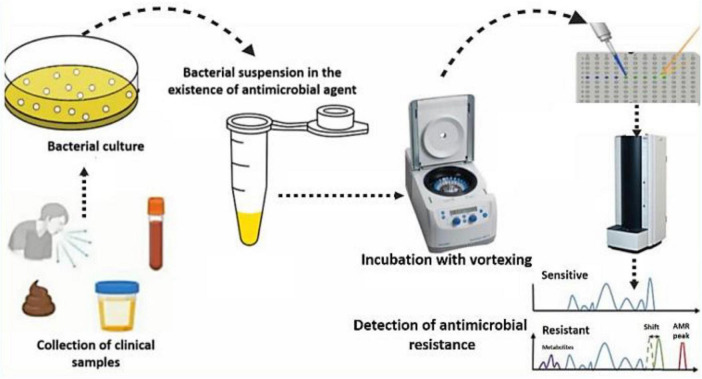
A workflow for the detection of antimicrobial resistance (AMR) using matrix-assisted laser desorption ionization-time of flight mass spectrometry (MALDI-TOF MS).

Peptide fingerprinting analytical technique (PFAT) can be used to identify proteins in microorganisms, including bacteria and fungi (Elbehiry et al., 2022a; Elbehiry et al., 2022c; Elbehiry et al., 2023). It breaks down target proteins into smaller peptides and measures the mass-charge ratio (m/z), such as matrix-assisted laser desorption ionization-time-of-flight mass spectrometry (MALDI-TOF MS). This method is valuable for assessing AMR (Figure 2), bridging the gap between species identification and resistance evaluation (Florio et al., 2020; Vrioni et al., 2018). Since 2010, healthcare settings have utilized MALDI-TOF MS, which is easier, faster, more accurate, and less expensive than traditional biochemical methods (Elbehiry et al., 2022a). The San Lazaro Hospital-Nagasaki Collaborative Research Laboratory analyzed approximately 13,000 bacterial and fungal isolates via MALDI-TOF MS over a span of 5 years (Osa et al., 2021). This system is able to detect resistance mechanisms and identify biomarkers, making it vital for monitoring resistant infections and facilitating rapid diagnosis (Rodríguez-Sánchez et al., 2019). Compared with traditional methods, MALDI-TOF accelerates resistance identification (Vrioni et al., 2018). The MBT-ASTRA evaluates bacterial growth by comparing the area under the curve (AUC) of bacterial peaks in antibiotic-treated (AUC_*BAC*+*ATB*_) versus untreated (AUC_*BAC*_) spectra. The ratio (AUC_*BAC*+*ATB*_)/(AUC_*BAC*_) is known as relative growth (RG). An RG close to zero indicates susceptibility, whereas an RG near one suggests resistance (Lange et al., 2014; Sparbier et al., 2016).

Matrix-assisted laser desorption ionization-time of flight mass spectrometry has shown potential for detecting antibiotic resistance in clinical samples, but its use is limited by low sensitivity and a focus on relatively small resistance pathways (Yamin et al., 2023). Recent machine learning advancements have improved the ability of these methods to recognize drug-resistant bacteria (Griffin et al., 2012). MBT-ASTRA quickly analyzes spectra, providing AST results within 2–3 h (Vrioni et al., 2018). A study conducted by Rhoads et al. (2016) evaluated the utility of the 2,415 m/z peak in clinical isolates of *S. aureus* and *Staphylococcus epidermidis* as a predictor of *mecA* gene carriage. Although numerous molecular assays are available for the rapid detection of *mecA*, these tests are predominantly performed on blood cultures. Nevertheless, in certain staphylococcal species, MALDI-TOF MS may serve as an effective and expedient method for the detection of *mecA* carriage. Timely diagnosis of *mecA* is crucial for facilitating appropriate medical intervention and enhancing antibiotic stewardship. By analyzing data obtained from routine MALDI-TOF MS testing, it is feasible to identify methicillin resistance in a subset of clinically isolated staphylococci. A recent study conducted by Bianco et al. (2022a) in a tertiary teaching hospital in northwestern Italy over a 3 years period (November 2019–October 2022) revealed that the MALDI Biotyper^®^ platform can accurately and effectively identify *K. pneumoniae* carbapenemase *(KPC)* producers without the need for additional procedures or extra costs. This MALDI-based method is suitable for high-throughput laboratories. Depending on the local prevalence of non-pKpQIL-encoded KPC and other carbapenemases, molecular or immunochromatographic tests should be utilized.

The advantages of MALDI-TOF MS include precision, reliability, affordability in terms of cost/sample run, and simplicity (Vrioni et al., 2018). However, spectrum analysis software usually requires programming skills that many microbiology laboratory specialists do not possess (Welker and Van Belkum, 2019). The technique also requires purification and sample preparation, making it unsuitable for mixed materials, and relies on additional chemicals such as those in the matrix (Wang et al., 2018). Databases should differentiate between susceptible and resistant strains (Dubourg and Raoult, 2016). Mass spectrometry can identify key resistance genes, such as *vanA*, *blaKPC*, and *mecA* (Neil et al., 2021). Although Bruker Daltonics has created a prototype software package, it is not yet available (Wilhelm et al., 2023). Therefore, there is considerable interest in developing a modified version of this test for hospitals in need of faster AST results.

### 2.4 Future techniques

#### 2.4.1 Hybridization-based systems (fluorescence in situ hybridization)

The fluorescence in situ hybridization (FISH) technique is effective for quantifying target microorganisms (Swidsinski et al., 2022; Vasala et al., 2020). PNA-FISH, which uses peptide nucleic acid (PNA) probes, offers rapid and specific binding advantages over DNA or RNA probes (Almeida et al., 2009; Vasala et al., 2020). Its electrically neutral backbone minimizes non-specific attachment, making it reliable for routine diagnostics, although PNA probes are often more expensive. Enroth et al. (2019) highlighted the use of PNA-FISH in QuickFish technology (OpGen, United States), which targets *16S rRNA* for identification. FISH-based identification of resistance determinants is a fast, simple, and affordable method. Many AMR genes, such as *bla*_*CTX–M*_ (Khan et al., 2020), *mecA* (Zhu and Li, 1994), and *vanA* (Conwell et al., 2021), can be detected via FISH. Salimnia et al. (2014) revealed that XpressFISH can identify the *mecA* gene in MRSA alongside QuickFish, allowing the diagnosis of methicillin resistance within 2 h of a positive blood culture. FISH can be used to assess resistance directly from raw materials such as tissues, making it suitable for resource-limited settings. Two conditions must be satisfied for FISH to be effective in identifying resistance determinants. First, the high abundance of ribosomal RNA copies in living cells results in ribosomally mediated resistance, which influences the efficacy of antibiotics such as macrolides and linezolid and is particularly suitable for FISH analysis. Second, FISH can be effectively employed when resistance is conferred by only one or a few variable bases, thereby eliminating the necessity for a large array of probes (Frickmann et al., 2014).

Unlike PCR, FISH can pinpoint specific resistance mechanisms in bacteria, but its applications are limited, and the results may be inconsistent (Frickmann et al., 2014). Resistance testing with FISH lacks standardization, posing challenges. Additionally, tissue autofluorescence must be considered, requiring expert interpretation of the results (Vasala et al., 2020). Expertise is required to interpret outcomes, especially when dealing with tissue autofluorescence. Counterstaining with a paneubacterial FISH probe and non-specific DNA stains is crucial for confirming nucleic acids associated with detected illnesses (Swidsinski, 2006). Owing to its limitations, FISH may serve as a temporary solution until more user-friendly and cost-effective amplification-based alternatives become available (Frickmann et al., 2014). Future research that investigates the mechanisms underlying drug resistance and the failure of eradication efforts in developing countries, particularly regarding clarithromycin and its related medications, may benefit from the application of the FISH approach (Frickmann et al., 2017).

#### 2.4.2 DNA microarray (DNA chip) technique

Microarray analysis is a valuable method for examining genomic AMR in microorganisms (Waskito et al., 2022). It allows for the purification and amplification of specific RNA molecules, facilitating gene expression analysis and gene function determination (Galhano et al., 2021; Hung and Weng, 2017). Microarrays are effective in gene transcriptome studies and can identify AMR genes via multiple hybridization procedures on the same substrate. DNA samples can be extracted without culturing bacterial cells (Nsofor, 2014). However, microarrays are increasingly being replaced by next-generation sequencing (Galhano et al., 2021). A key limitation is the requirement for prior knowledge of the genetic region being studied, which may result in overlooked data (Kanzi et al., 2020). Additionally, analyzing target genes can be complicated because of hybridization of similar sequences (Jaksik et al., 2015).

Baumgartner et al. (2014) used the microarray method to identify genes linked to resistance in foodborne pathogens and reported that *mecA*, *vanB*, *msr*, *aadD*, and *cat* confer resistance to methicillin, vancomycin, macrolides, tobramycin, and chloramphenicol, respectively. Vogt et al. (2014) reported strains of *E. coli* resistant to third-generation cephalosporins in various meats and identified genes associated with resistance to trimethoprim (*dfrA*), sulfonamides (*sul*), tetracycline (*tet*), and chloramphenicol (*cmlA1*-like). Conventional resistance tests for *Mycobacterium tuberculosis* are time-consuming, delaying treatment (Asmare and Erkihun, 2023). Microarray analysis can quickly identify AMR by detecting mutations in the *rpoB* and *katG* genes linked to rifampin and isoniazid resistance (Herrera-Rodriguez et al., 2013). It can also identify ESBL- and carbapenemase-producing Enterobacteriaceae (Dortet et al., 2014). Microarrays are more effective and faster at detecting AMR genes in both gram-positive and gram-negative microbes (Fink et al., 2019). Recently, microarrays have emerged as widely utilized diagnostic techniques in biological research and clinical laboratories around the world (Rabiee et al., 2021). Song et al. (2019) analyzed 416 clinical samples from the Chinese PLA General Hospital via microarray technology, which directly identified eight carbapenemase genes across diverse samples. This method is recognized for its user-friendly design, adaptability for high-throughput detection, and clinical application potential. In 2019, Fink et al. (2019) evaluated a DNA microarray for the detection of AMR genes in 240 g-positive and gram-negative bacterial isolates. These genes included plasmid-encoded extended-spectrum β-lactamases and carbapenemases, as well as *mecA*, *vanA*, and *vanB*. The isolates were sourced from the Microbiology Services of two hospitals in Valencia, Spain. The assay demonstrated 100% sensitivity and specificity for all target genes. Although the manufacturing of DNA microarrays has advanced significantly and has been commercialized in recent decades, the constraints associated with high-resolution scanning, along with the time-intensive and expensive processes involved in microarray production, continue to restrict their accessibility to many laboratories (Xu et al., 2024).

#### 2.4.3 Flow cytometry laser-based technique

Flow cytometry, introduced in the 1960s, is an effective single-cell analytical method that has significantly impacted fields such as immunology, molecular biology, chemotherapy, healthcare microbiology, ecological microbiology, and the food and beverage industries (Marutescu, 2023). Advances in cytometry have transformed life sciences and biomedicine by enabling rapid evaluation of bacterial cells without cultivation (Robinson et al., 2023). Researchers are developing strategies and tests to combat AMR, using flow cytometry to identify antibiotic-resistant microorganisms in healthcare and environmental samples (Godeux et al., 2018; Zhang et al., 2023). Flow cytometry analysis in healthcare can assess a microorganism’s susceptibility, resistance, or intermediate status to antibiotics (Martins-Oliveira et al., 2020; Silva-Dias et al., 2021). It quickly identifies AMR patterns in hospital samples (Filbrun et al., 2022; Inglis et al., 2020; Mulroney et al., 2022; Velican et al., 2020) and uses fluorescent dyes to evaluate bacterial cell viability after antibiotic treatment.

In accordance with the recommendations of Thermo Fisher Scientific, the flow cytometry-AST workflow comprises several critical steps. Initially, bacteria are collected and suspended in an appropriate solution to minimize background noise for the Attune (NxT) flow cytometer. The bacteria are subsequently exposed to various antibiotics, and a vital dye is applied to facilitate detection. The preinstalled software then analyzes the gated populations, monitoring alterations in size, shape, and color intensity induced by the antibiotics. The Attune (NxT) flow cytometer effectively illustrates the impact of varying antibiotic concentrations on these parameters, rendering this method more efficient than traditional culture-based MIC determination. However, limitations exist, such as non-specific dye binding, variable microorganism–antimicrobial interactions, and inadequate computational power for diverse populations (Waagsbø et al., 2022). Recently, commercial flow cytometry-based ASTs have been introduced, providing results in under 2 h, whereas traditional methods require 24–48 h (Martins-Oliveira et al., 2020; Silva et al., 2019; Silva-Dias et al., 2021). Unlike conventional culture-based methods, flow cytometric tests can determine microorganism fatality in medical samples treated rapidly with pharmaceuticals in less than 24 h (Marutescu, 2023).

Flow cytometric assays assess bacterial susceptibility, resistance, or intermediate responses to antibiotics in clinical microbiology. In 2020, Velican et al. (2020) evaluated the antibiotic susceptibility of 29 *E. coli* strains from urine samples of hospitalized patients in Bucharest, Romania. The technique effectively quantified susceptibility to nitrofurantoin, trimethoprim-sulfamethoxazole, ciprofloxacin, and ceftriaxone. In 2021, Kállai and his team at Budapest (Kállai et al., 2021) developed AST protocols using flow cytometry on six bacterial strains: *E. coli*, *K. pneumoniae*, *P. aeruginosa*, *S. aureus*, *Streptococcus pyogenes*, and *Enterococcus faecalis*. A flow cytometer was used to monitor bacterial growth to determine the optimal AST timing. The bacteria were tested against 12 antibiotics, with MIC values compared with microdilution values as a reference. Using EUCAST clinical breakpoints, susceptibility profile-matched microdilution results in more than 92% of the cases, indicating that flow cytometry-AST is an efficient method for generating susceptibility profiles with a low failure rate. Costa-de-Oliveira et al. (2017) studied the effectiveness of FASTinov^®^ testing for managing gram-negative bacteremia at Centro Hospitalar S. João in Porto, Portugal, in 2015. An analysis of 102 positive blood cultures via routine methods, the Vitek2 test, the FASTinov^®^ kit, and the gold standard microdilution method revealed that the FASTinov^®^ kit yielded significantly faster results than did the Vitek2 test or the standard method. Although molecular ASTs are faster and can predict phenotypic resistance, varying sensitivity levels necessitate phenotypic screening to identify emerging resistance pathways (Marutescu, 2023).

#### 2.4.4 Fourier transform infrared (ftir) spectroscopy

Microbiology laboratories and healthcare settings have greatly benefited from advancements in optical techniques (Kaprou et al., 2021), particularly infrared (IR) spectroscopy and microscopy, which enhance the collection of molecular-level biological data on microbes (Beć et al., 2020). Fourier transform infrared spectroscopy (FTIR) is an innovative tool for biochemical evaluation that provides detailed information on a substance’s chemical composition (Chirman and Pleshko, 2021; Theakstone et al., 2021). By measuring infrared light absorption by proteins, carbohydrates, lipids, and genetic material, FTIR generates a spectrum that accurately reflects the sample’s composition (Candoğan et al., 2021; Vogt et al., 2019). Its application is increasingly used to identify chemical changes linked to AMR in prokaryotic organisms (Novais et al., 2019; Salman et al., 2017).

In 2017, Sharaha et al. (2017) used FTIR to assess antibiotic susceptibility in bacteria through IR spectroscopy. After 24 h of culturing bacterial colonies from patient samples, they employed a computer classification method and an IR microscope to evaluate *E. coli* sensitivity to gentamicin, ceftazidime, nitrofurantoin, nalidixic acid, and ofloxacin. Approximately 85% of bacteria are classified as susceptible or resistant, highlighting the technique’s potential for rapid testing. Abu-Aqil et al. (2024) employed FTIR spectroscopy in conjunction with machine learning techniques to identify 636 strains of *K. pneumoniae* from urine samples collected from patients with urinary tract infections and to assess their antibiotic susceptibility. An analysis of a total of 27,966 spectra via an XGBoost classifier revealed that the identification accuracy exceeded 95%, with the sensitivity for antibiotic susceptibility ranging from 74 to 81%. These results suggest that the proposed system has the potential to reduce the risks associated with bacterial resistance; however, further research is necessary to validate these findings. Research by Kochan and colleagues indicated that *S. aureus* has evolved chemically, leading to resistance against daptomycin and vancomycin. They utilized atomic force microscopy with infrared spectroscopy, a novel single-cell nanoscale technique (dos Santos et al., 2023; Li, 2023), which enhances the precision and imaging of atomic-scale structures within cells.

In 2022, Wang-Wang et al. (2022) assessed FTIR as a primary tool for identifying extended-spectrum β-lactamase-producing *K. pneumoniae* outbreaks in hospitals. Compared with traditional methods, FTIR identified more true clustering relationships (38 out of 42 vs. 24 out of 42, *p* = 0.001), highlighting its potential as a rapid and cost-effective detection tool, with WGS for confirmation. This integration can enhance infection control in clinical microbiology laboratories. FTIR provides advantages in AMR research, such as speed, cost-effectiveness, reliability, and environmental friendliness (Kaprou et al., 2021; Mashhadikhan et al., 2022; Yamin et al., 2023). However, its application in resource-limited settings as a point-of-care tool for testing AMR is hindered by the instrument’s size and cost (Kaprou et al., 2021). Accurate results require prioritizing sample purification, culturing, and processing, along with accessible datasets that distinguish between sensitive and resistant isolates.

#### 2.4.5 Lab-on-a-chip technology and microfluidics

**FIGURE 3:**
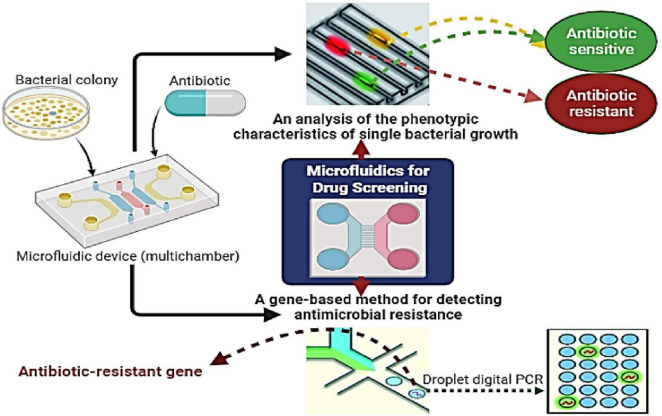
Procedures for the rapid screening of individual bacteria utilizing microfluidics encompass gene-based detection of antimicrobial resistance (AMR) through droplet digital assessment, as well as phenotypic evaluation via microfluidic-based single bacterial culture.

Microfluidics-based lab-on-a-chip technologies benefit various fields, including environmental surveillance (Fernandez-Gavela et al., 2019), food security (Tsougeni et al., 2019), and healthcare diagnostics (Papadakis et al., 2019), and have recently enabled the identification of antibiotic-resistant microorganisms (Li et al., 2020). MIC values are essential for analyzing bacterial phenotypic resistance, assessing new antimicrobial agents, and monitoring global drug resistance (Zhang et al., 2020). This technique determines if an antibiotic can inhibit pathogen growth, even if slowly. However, this delay prolongs the selection of effective treatments, leading to worse clinical outcomes and higher patient mortality rates (Li et al., 2017). To address this issue, developing technologies for the early detection of antibiotic resistance during therapy is crucial. In 2018, Zhang et al. (2018) developed a microfluidic chip platform to identify bacteria and detect antibiotic resistance genes in 108 cerebrospinal fluid culture broths from Beijing Tiantan Hospital and Capital Medical University, China. The platform achieved a 94.44% concordance rate with traditional methods and demonstrated over 90% sensitivity and specificity for carbapenemase and ESBL resistance genes. The study concluded that the platform is rapid, accurate, and user friendly, with strong potential for treating postneurosurgical meningitis. Ardila et al. (2023) conducted a systematic review examining the potential clinical applications of microfluidics-based lab-on-a-chip technology for the identification and assessment of antibiotic susceptibility in *Enterococcus faecalis* associated with endodontic infections. Although these platforms demonstrate significant potential for enhancing the diagnosis and treatment of *Enterococcus faecalis*, their effective implementation in clinical practice requires comprehensive research, development, and validation to ascertain their efficacy and reliability. Figure 3 shows recent microfluidic techniques for quick bacterial ASTs, including genotypic methods (such as droplet digital technology for resistance detection) and phenotypic methods (such as microfluidic single bacterial culture).

Lab-on-a-chip technologies offer advantages over macroscale methods, including rapid output evaluation, precise fluid control, cost-effectiveness, the utilization of minimal amounts of reagents/samples/solvents (few μL), automation, mobility, and process integration (Fernandez-Gavela et al., 2019). Microfluidic identification techniques fall into two categories: phenotypic and molecular analyses. Loop-mediated isothermal amplification enables rapid results by analyzing genetic markers such as AMR genes (Zhang et al., 2018). Integrating nucleic acid detection assays into microfluidic devices provides a cost-effective solution for diagnostics in the clinic, food safety, and environmental monitoring. These devices, which are designed for resource-limited settings and point-of-care use, require amplification of target nucleic acids for sensitive detection. Traditional PCR methods are limited by temperature cycling. Therefore, novel isothermal amplification techniques will be developed to integrate sample preparation and target identification using minimally invasive samples such as saliva, blood, or urine (Giuffrida and Spoto, 2017; Zhang et al., 2019).

Phenotypic microfluidic methods reliably track microorganism growth for AST in the presence of antimicrobial agents (Campbell et al., 2016). These methods utilize small amounts of microorganisms in droplets, channels, or chambers (Needs et al., 2020) and can encapsulate them in agarose-filled capsules or immobilize them using antibodies on magnetized substrates (Dong and Zhao, 2015). Hydrodynamic capture is one technique for immobilization, offering dense trap arrays and easy integration, but it has low effectiveness (Kaprou et al., 2021). Antibodies are costly and may not be accessible for all bacteria, whereas droplet-based methods often require complex reading techniques. Although agarose-based methods can use standard multiwell plates, they complicate automatic identification and information processing (Kaprou et al., 2021). Further research is needed before these devices can be commercially viable.

#### 2.4.6 Lateral flow immunoassays

The rise of antimicrobial-resistant bacteria has prompted the development of lateral flow immunoassays (LFIAs) that target enzyme-mediated resistance in key pathogens (Boutal et al., 2022). Despite advancements, identifying pathogenic bacteria and detecting antibiotic resistance remain complex (Shanmugakani et al., 2020). In the last decade, LFIAs have improved the diagnosis of antimicrobial resistance, proving essential for detecting resistance mechanisms in gram-negative bacteria, especially beta-lactamases (Bernabeu et al., 2020; Boutal et al., 2018; Han et al., 2021). One LFIA identified the AAC (6’)-Iae enzyme from *P. aeruginosa* with a sensitivity of 10^5 CFU per test (Kitao et al., 2010). Another LFIA targets the ArmA 16S rRNA methylase, which is prevalent in resistant gram-negative bacteria such as *A. baumannii* and *E. coli* (Oshiro et al., 2015). Early identification of methicillin resistance relies on detecting penicillin-binding protein 2a (PBP2a), which affects all beta-lactams. The affinity of *S. aureus* protein A for mammalian immunoglobulins complicates antibody-based detection. Nevertheless, Yamada et al. (2013) used an LFIA, whereas Amini et al. (2020) utilized IgY anti-PBP2a antibodies. For *VanA* vancomycin-resistant *Enterococcus* (VRE) isolates, LFIA is 100% sensitive and specific, with detection limits of 6.3 × 10^6 or 4.9 × 10^5 cfu per test on either MH or ChromID^®^ VRE plates, respectively (Oueslati et al., 2021).

Lateral flow immunoassays have been developed for the detection of KPC and OXA-48-like enzymes (Coris Bioconcept, Gembloux, Belgium) (Wareham et al., 2016), as well as IMP, which is recognized as the most prevalent metallo-β-lactamase in Japan (Notake et al., 2013), and New Delhi metallo-beta-lactamase 1 (NDM) (Boutal et al., 2017; Tada et al., 2019). These assays demonstrate 100% sensitivity and specificity when utilized with isolated colonies obtained from agar plates. Comini et al. (2022) evaluated a rapid diagnostic method using MALDI-TOF MS, LFIAs, and molecular testing to identify gram-negative bacteria and key β-lactamases from positive blood cultures. The NG-Test^®^ CARBA 5 and NG-Test^®^ CTX-M MULTI LFIAs had sensitivities of 92.2 and 91.6%, respectively, whereas the Easyplex^®^ SuperBug CRE showed 100% sensitivity for *bla*_*KPC*_ mutations linked to ceftazidime/avibactam resistance. No false positives were found, making this method a cost-effective solution for quickly identifying gram-negative organisms and resistance indicators. In a French clinical laboratory, the NG-Test CTX-M MULTI identified 98% of ESBL producers from colonies and positive blood cultures (Bernabeu et al., 2020). While CTX-M enzymes are primarily responsible for extended-spectrum cephalosporin (ESC) resistance, some uncommon enzymes, such as plasmid-encoded AmpC and certain carbapenemases, may not be detected. The LFIA-CTX test showed 99.1% sensitivity and 100% specificity for detecting ESC hydrolytic activity in colonies (Moguet et al., 2022).

A recent evaluation of ESC hydrolysis compared three assays: the ESBL NDP test, the ZG-Lacta™ test, and the LFIA-CTX test with the NG-Test CTX-M-Multi. LFIA-CTX demonstrated increased sensitivity and specificity, particularly for Enterobacterales. LFIA-CTX offers the advantage of detecting more than just ESBLs, unlike the NDP test. Its integration with NG-CTX-M-Multi effectively enables the hydrolysis of extended-spectrum cephalosporins (ESCs) and ESBLs. Moguet et al. (2022) introduced a novel LFIA strip that combines the LFIA-CTX and NG-CTX-M-Multi assays. This strip allows clinical microbiology laboratories to detect extended-spectrum cephalosporin (ESC) hydrolysis directly from colonies grown on culture media used for CTX-M-like enzymes, which represent 98% of ESBLs. This improvement in antimicrobial stewardship allows for early treatment with new medications. NG Biotech’s LFIA can detect MCR-1 within 15 min via the use of streptavidin-labeled anti-MCR-1 mouse monoclonal antibodies. A multicentric validation study revealed that this test successfully identified 109 true-positive MCR-1 cases with no false-negative results. Additionally, the assay detected three *E. coli* isolates that produce MCR-2. In contrast, isolates producing MCR-3, MCR-4, or MCR-5 tested negative (Smelikova et al., 2022; Volland et al., 2019).

#### 2.4.7 CRISPR-Cas system antimicrobials

A novel strategy is urgently needed to address the rise of antibiotic resistance and infectious diseases caused by bacteria. The CRISPR/Cas system, an RNA-guided adaptive immune mechanism in prokaryotes, identifies and neutralizes invasive genetic elements such as plasmids and phages (Tao et al., 2022). This technology is being developed to prevent and control antimicrobial resistance, effectively targeting DNA sequences that carry resistance genes (Aslam et al., 2020; Duan et al., 2021; Wu et al., 2021). Over the past few decades, CRISPR/Cas has become increasingly popular for gene modification. Some research suggests that it could be more effective in addressing antibiotic resistance genes in certain cell types and experimental platforms (Manghwar et al., 2019). Antibiotic-resistant bacteria pose a threat to human health and hinder the progress of modern medicine (Okaiyeto et al., 2024). By swiftly and accurately identifying resistance, healthcare practitioners can administer appropriate treatment faster, optimize the use of current antibiotics, and avoid resorting to “last resort” medications (Raro et al., 2024). Therefore, the development of technology capable of rapidly and accurately detecting drug resistance genes is crucial for the advancement of modern medicine. Since its discovery in 2012 in *Streptococcus pyogenes*, the Cas9 protein in the CRISPR system has led to the development of numerous novel applications (Jinek et al., 2012). The detection of antibiotic resistance genes and bacterial infections has also become increasingly prevalent in recent years.

Müller et al. (2016) developed an optical DNA mapping technique utilizing a single plasmid within a nanofluidic tube. This method leverages the cleavage capabilities of Cas9 to convert a circular plasmid into a linear form, thereby facilitating the detection of resistance genes. The CRISPR/Cas9 system incorporates a gene-specific RNA (gRNA) that assists in the identification of target genes. The optical DNA mapping technique can identify this gRNA by linearizing it at a predetermined location on the circular plasmid. Through their investigations, researchers have examined the potential for enhancing this detection technology for future clinical applications. They integrate multiple gRNAs that target various genes associated with antibiotic resistance. In a study conducted by Nyblom et al., strains of *E. coli* and *K. pneumoniae* were directly identified in patient samples via this approach (Nyblom et al., 2023). By targeting antibiotic resistance genes with the Cas9 protein, researchers have successfully identified specific strains or subtypes, as well as plasmids associated with these strains. This optical DNA profiling technique enables the rapid acquisition of comprehensive diagnostic information, thereby optimizing antibiotic treatment regimens and paving the way for the future of precision medicine management.

In 2023, (Qin et al. (2023). developed a method using CRISPR/Cas9-induced isothermal exponential amplification reactions (IEXPARs) to discover antibiotic resistance genes. They achieved this by cleaving the genes into two short fragments with free 3’-OH ends. The cleaved DNA templates trigger exponential amplification of IEXPAR under isothermal conditions, enabling the direct detection of antibiotic resistance genes. This rapid and precise method relies on the CRISPR/Cas9 system, which cleaves DNA only in the presence of antibiotic resistance genes. This ensures high specificity, as antibiotic-sensitive bacteria lack these genes. After approximately half an hour of amplification, the method has a detection limit of 81 fM, allowing for the identification of antibiotic resistance genes at concentrations as low as 100 fM. This method is effective in detecting both antibiotic-resistant and antibiotic-sensitive bacteria in real biological samples under isothermal conditions.

A study conducted in 2023 by Gao et al. (2023) introduced a CRISPR/Cas12a-based colorimetric paper sensor. This sensor utilizes the trans-cleavage activity of Cas12a, which is enhanced through rolling circle replication, to generate a 3D DNAzyme that strongly adheres to the paper. This resulted in a sensor with a high concentration of functional DNAzymes, making it highly bioactive. The assay was designed to swiftly detect the antibiotic resistance gene NDM with exceptional sensitivity. In the absence of the gene, the 3D DNAzyme produced a blue color. Conversely, the presence of the gene triggered collateral Cas12a cleavage activity, causing the circular template to be cleaved and preventing the formation of the 3D DNAzyme, leading to no colorimetric signal. This paper introduces a sensor capable of rapidly and affordably detecting antibiotic resistance genes in pathogenic microorganisms with femtomolar sensitivity. The results are visible to the naked eye, and the analysis can be completed in under one and a half hours. The programmable CRISPR probe design shown in this study has potential for swift responses to emerging global epidemics. At present, CRISPR-based antimicrobials are not widely utilized, so there have been no reports of resistance to this type of antimicrobial in clinically significant bacteria (Mayorga-Ramos et al., 2023). Research indicates that CRISPR-Cas systems could serve as next-generation antimicrobials, although their clinical application remains largely unexplored. Concerns regarding the safety and environmental implications of CRISPR-Cas technology highlight the need for risk assessments and strict regulatory measures for therapeutic purposes (Caplan et al., 2015). It is crucial to engage and educate the medical community and the public about this emerging technology to ensure its responsible and safe utilization (Javed et al., 2018; Pursey et al., 2018).

## 3 Final thoughts and future prospects

Collaboration among scientists, risk administrators, government agencies, and businesses is essential to address the AMR crisis and improve ASTs. Current instruments have limitations, such as the need for specimens before therapy and a lack of integration and mobility. Extensive biological methods remain the only viable options for infection detection. Innovative screening technologies are essential for expediting approval and commercialization. They should provide improved accuracy, faster turnaround times, lower costs, enhanced accessibility, and scalability in various healthcare settings. Faster and more reliable detection will benefit both healthcare practitioners and patients. Investing in the improvement of existing procedures and tools is necessary. A MarketsandMarkets Research Pvt. An Ltd. report predicts that the AST market will reach USD 4.2 billion by 2025 (Kaprou et al., 2021). However, the high costs of programmed AST systems limit their use, especially among budget-constrained organizations, despite their effectiveness in reducing incubation and identification periods (MarketsandMarkets™, 2021). In 2019, manual AST devices were popular because of their lower costs, with the disk diffusion method holding the largest market share due to the variety and affordability of disks. Healthcare facilities and testing laboratories account for the largest segment of the AST market (MarketResearch.com, 2021). AST has been proven to reduce hospital stays and improve patient outcomes (Cassini et al., 2019). However, the cost of molecular testing, which ranges from $100–$250 per test (Li et al., 2017), presents a significant barrier to its widespread implementation. Additionally, NAATs can result in annual reagent costs exceeding $500,000 for a 500-bed community hospital (She and Bender, 2019). In contrast, AST using mass spectrometry costs approximately €79 per patient, with reagent costs of approximately $1 (Patel et al., 2017). The costs associated with Microscan and Vitek susceptibility testing typically range between €30 and €50 (European Union, 2013). The variability in pricing for different AST methodologies further complicates the estimation of overall expenses (Vasala et al., 2020).

While the methods and technologies mentioned here show promise in addressing antimicrobial resistance, several questions remain. What percentage are consistently effective? Do they meet industry standards? When will new techniques be widely available? Although many claims to detect resistance within hours, they often neglect time-consuming steps such as culture isolation and enrichment before treatment. The traditional AST takes 18 to 36 h and provides MICs (Gajic et al., 2022); however, it is unsuitable for non-culturable microorganisms. The automated systems currently available do not accommodate these organisms and have response times of 2–24 h. Some methods can measure MICs (Kasas et al., 2021), while MALDI-TOF MS can yield results in 2–4 h, although it shares limitations with traditional methods and lacks processing software (Elbehiry et al., 2022a). NAATs can be used with ASTs when culture is not possible (Johnson et al., 2002; Theel and Schuetz, 2022), and new AMR genes can be detected. WGS is a promising tool for rapid AST but requires extensive datasets, making bioinformatics a major challenge (Bogaerts et al., 2019). An overview of the benefits and drawbacks of these techniques is provided in Table 1.

**TABLE 1:** An analysis of the benefits and drawbacks of conventional and non-conventional methods used to detect antimicrobial resistance.

Technique	Benefits	Drawbacks
**Traditional techniques**		
Phenotypic	This technique is unique due to its simplicity and verifiable methods, allowing it to serve as a reference, determine the MIC, and identify pathogens.	The guidelines vary, requiring prior cultivation, which is challenging for fastidious microbes and impossible for non-cultivable ones.
Molecular	It allows for the analysis of polymicrobial specimens, multiplexing of resistance determinants, and quick detection of resistance genes, facilitating a rapid response to emerging resistance factors.	AMR genes present challenges such as the need for specialized training, high laboratory costs, difficulties in defining MIC, and the risk of overlooking certain genes.
**Techniques currently being utilized**		
Next-generation sequencing	The ability to identify fastidious, non-cultivable microbes is improved by long-read sequencing devices, which are convenient and resource-efficient. Genome sequencing characterizes new resistance mechanisms and establishes the genetic basis of antimicrobial resistance, enabling simultaneous investigation of resistance determinants from various hosts through whole metagenomic sequencing.	High equipment costs, labor-intensive methods, and the need for trained personnel. The unidentified hosts of AMR determinants complicate the determination of the MIC, and the link between phenotypic resistance and MIC is not fully understood.
MALDI-TOF mass spectrometry	Rapid investigation, high productivity, automated techniques, low costs, small sample sizes, and screening of antimicrobial resistance.	Requires prior cultivation and significant equipment costs. Libraries must include spectra from both sensitive and resistant strains. AMR biomarkers face challenges such as limited mobility, statistical differentiation, and determining the MIC.
**Future techniques**		
DNA microarray (DNA chip) technique	Identifying multiple resistance genes simultaneously enhances AMR detection. This rapid, high-throughput method is crucial for large-scale surveillance of multidrug-resistant organisms and for addressing AMR.	High costs, and complicated data interpretation. In clinical practice, they work best when combined with antibiotic susceptibility testing.
Flow cytometry laser-based technique	This method enables high-throughput, simultaneous real-time antibiotic susceptibility testing, efficiently detecting phenotypic resistance and mechanisms.	Requires technical expertise, is costly, complicates the identification of new resistance mechanisms, and lacks quantitative antibiotic susceptibility data, such as MIC values.
FTIR spectroscopy	Rapid examination, high-throughput automation, easy sample handling, low costs, and minimal specimen volume.	Prior cultivation, database establishment (including spectra from resistant strains), and the acquisition of AMR biomarkers are essential. However, these patterns are not applicable to all microbes and require mathematical skills and discrimination processes.
Fluorescence in situ hybridization (FISH)	Rapid detection of AMR genes in clinical samples without culturing, and its multiplexing and visualization capabilities improve the study of antimicrobial resistance.	FISH faces challenges such as reliance on known resistance genes, potential false positives, and insufficient phenotypic susceptibility data. Its complexity and need for specialized equipment limit its practicality in resource-poor settings and large-scale surveillance.
Lab-on-a-chip and microfluidics	Rapid analysis delivers high productivity, precise fluid handling, low costs, minimal reagent and energy use, small sample sizes, automation, and easy sample preparation.	Challenges include defining MIC, scalability and reproducibility issues in fabrication, a large surface-to-volume ratio, commercialization hurdles, and the need for surface treatments to reduce adsorption.
Lateral flow immunoassays	The implementation of these methods offers several advantages in combating antimicrobial resistance, especially in terms of their speed, cost-effectiveness, and user-friendliness in resource-limited environments. They are particularly beneficial for quickly screening specific resistance markers and for use in large-scale surveillance programs.	The inability of these methods to identify specific resistance patterns, their limited applicability, and lack of comprehensive profiling make them unsuitable for complex or high-stakes cases that require precise knowledge of resistance.
CRISPR-Cas-based detection methods	Detection of antibiotic resistance and virulence genes, diagnosis of bacterial infections, identification of genotypes and single nucleotide polymorphisms, high specificity and sensitivity, and time-saving.	This technique encounters challenges, such as a high incidence of bacterial escape from double-stranded DNA breaks induced by CRISPR-Cas systems and alterations in the CRISPR-Cas effector protein, guide RNA (gRNA), or target sequence.

Meeting the demands for rapid ASTs is challenging, and no existing methods are ideal. However, some may significantly influence the quick AST market. This market includes point-of-need services offered by well-equipped labs in healthcare and research facilities, which can integrate methods such as whole-genome sequencing, PCR, and automated AST systems. Alternatively, portable microfluidic AST systems may be better suited for smaller labs and medical professionals, offering cost-effectiveness, mobility, and quick turnaround times.

## 4 Conclusion

Identifying AMR is crucial in combating resistant infections. Advances in diagnostic technology have revolutionized AMR identification and treatment. While traditional methods such as disk diffusion and MIC testing are accessible and cost-effective, they often lack the speed and sensitivity required to detect new resistance mechanisms. Modern technologies, such as CRISPR-based systems, PCR diagnostics, MALDI-TOF mass spectrometry, and next-generation sequencing (NGS), have significantly improved AMR diagnosis by enabling comprehensive genomic analyses and providing real-time results. However, challenges such as cost, complexity, and the need for specialized training persist. The future of AMR detection will likely involve a combination of traditional and modern methods, allowing for quicker and more accurate diagnostics. This integrated approach can improve treatment decisions, enhance surveillance, and ultimately lead to better patient outcomes in the global fight against antimicrobial resistance. Continued research, investment in infrastructure, and policy support are essential for the effective clinical application of these advancements.
